# Crystal structure of bis­[*N*-(2-hy­droxy­eth­yl)-*N*-methyl­dithio­carbamato-κ^2^ *S*,*S*′](pyridine)­zinc(II) pyridine monosolvate and its *N*-ethyl analogue

**DOI:** 10.1107/S2056989017010568

**Published:** 2017-07-21

**Authors:** Pavel Poplaukhin, Edward R. T. Tiekink

**Affiliations:** a Chemical Abstracts Service, 2540 Olentangy River Rd, Columbus, Ohio 43202, USA; b Centre for Crystalline Materials, Faculty of Science and Technology, Sunway University, 47500 Bandar Sunway, Selangor Darul Ehsan, Malaysia

**Keywords:** crystal structure, zinc, di­thio­carbamate, pyridine adduct, hydrogen bonding

## Abstract

The common structural feature of the title compounds, [Zn(C_4_H_8_NOS_2_)_2_(C_5_H_5_N)]·C_5_H_5_N (I) and [Zn(C_5_H_10_NOS_2_)_2_(C_5_H_5_N)]·C_5_H_5_N (II), which differ by having di­thio­carbamate N-bound methyl (I) and ethyl (II) groups, is the coordination of each Zn^II^ atom by two non-symmetrically chelating di­thio­carbamate ligands and by a pyridine ligand; in each case, the non-coordinating pyridine mol­ecule is connected to the Zn-containing mol­ecule *via* a (hy­droxy)O—H⋯N(pyridine) hydrogen bond. The resulting NS_4_ coordination geometry is closer to a square-pyramid than a trigonal bipyramid in the case of (I), but almost inter­mediate between the two extremes in (II). The mol­ecular packing features (hy­droxy)O—H⋯O(hy­droxy) hydrogen bonds, leading to supra­molecular chains with a zigzag arrangement along [10-1] (I) or a helical arrangement along [010] (II). In (I), π–π [inter-centroid distances = 3.4738 (10) and 3.4848 (10) Å] between coordinating and non-coordinating pyridine mol­ecules lead to stacks comprising alternating rings along the *a* axis. In (II), weaker π–π contacts occur between centrosymmetrically related pairs of coordinating pyridine mol­ecules [inter-centroid separation = 3.9815 (14) Å]. Further inter­actions, including C—H⋯π(chelate) inter­actions in (I), lead to a three-dimensional architecture in each case.

## Abstract 2

The Zn^II^ atoms in {Zn[S_2_CN(*R*)CH_2_CH_2_OH]_2_(pyridine)·pyridine}, for *R* = Me (I) and Et (II), are coordinated non-symmetrically by two di­thio­carbamate ligands and by a pyridine ligand, resulting in an NS_4_ donor set that defines a distorted geometry in each case; the non-coordinating pyridine mol­ecules are connected with the Zn-containing mol­ecules *via* O—H⋯N(pyridine) hydrogen bonds. The mol­ecular packing features significant (hy­droxy)O—H⋯O(hy­droxy) hydrogen bonding, in each case leading to supra­molecular chains with zigzag (I) or helical (II) arrangements.

## Chemical context   

Potentially multidentate ligands such as di­thio­carbamate, ^−^S_2_CN*RR*′, di­thio­carbonate (xanthate), ^−^S_2_CO*R*, and di­thio­phosphate, ^−^S_2_P(O*R*)(O*R*′), all belong to the 1,1-di­thiol­ate class of ligands. While many similarities are apparent in their coordination propensities (Hogarth, 2005 ▸; Heard, 2005 ▸; Tiekink & Haiduc, 2005 ▸; Haiduc & Sowerby, 1996 ▸), stark differences sometimes occur. As a case in point are species formed with the potentially bidentate ligand *trans*-1,2-bis­(4-pyrid­yl)ethyl­ene (bpe). With Zn(S_2_COEt)_2_, a 1:1 compound can be prepared which crystallography shows to be a one-dimensional coordination polymer with a zigzag arrangement (Kang *et al.*, 2010 ▸). A similar structure is found for the *R* = *n*-Bu species, but in the case of a bulky cyclo­hexyl (Cy) group only the dimeric aggregate [Zn(S_2_COCy)_2_]_2_(bpe) could be isolated (Kang *et al.*, 2010 ▸). Such steric control over supra­molecular aggregation is well established in the structural chemistry of main group 1,1-di­thiol­ate compounds (Tiekink, 2003 ▸, 2006 ▸). A similar situation to the above occurs for zinc(II) di­thio­phosphates, Zn[S_2_P(O*R*)_2_]_2_, in that 1:1 one-dimensional coordination polymers can be formed with bpe when *R* = *i*-Pr (Welte & Tiekink, 2007 ▸), *R* = *i*-Bu (Welte & Tiekink, 2006 ▸) and *R* = Cy (Lai *et al.*, 2004 ▸). Inter­estingly, when *R* is small, a zigzag chain is formed in the crystal but larger groups, *i.e. R* = Cy, lead to linear chains. The situation changes for zinc(II) di­thio­carbamates of bpe, where only binuclear species, [Zn(S_2_CN*R*_2_)_2_]_2_(bpe), have been isolated, *e.g. R* = Me (Poplaukhin & Tiekink, 2009 ▸), *R* = Et (Arman *et al.*, 2009*b* ▸) and *i*-Pr (Arman *et al.*, 2009*a* ▸). When an excess of bpe is introduced into the reaction with *R* = Et, the dimeric aggregate is again isolated and an additional mol­ecule of bpe is incorporated into the crystal (Lai & Tiekink, 2003 ▸). This contrasting behaviour can be explained in terms of an effective chelating mode of the di­thio­carabmate ligand owing to a 40% contribution of the ^2−^S_2_C=N^+^ *RR*′ canonical form to the overall electronic structure. This reduces the Lewis acidity of the zinc cation and results in an inability to increase its coordination number beyond five in these systems.
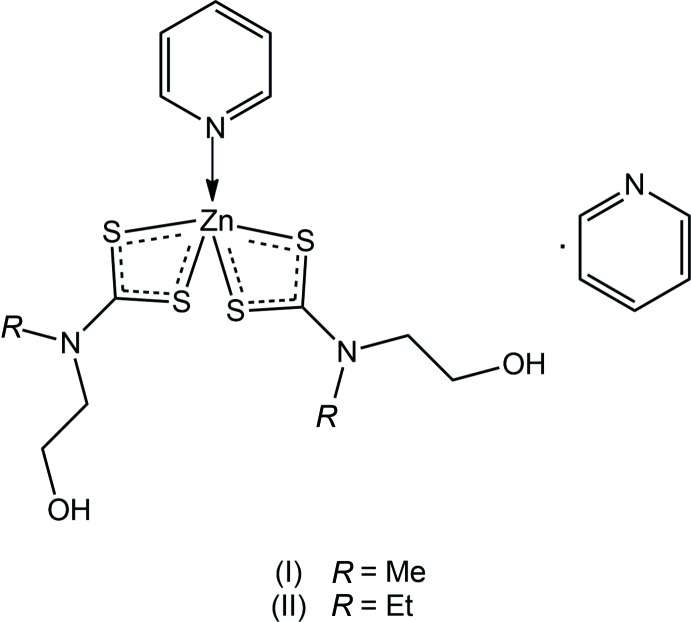


In order to overcome the reluctance of zinc(II) di­thio­carbamates to generate extended supra­molecular architectures, the di­thio­carbamate ligands can be functionalized with hydrogen-bonding potential, *i.e*. ^−^S_2_CN(*R*)CH_2_CH_2_OH (Howie *et al.*, 2008 ▸), and systematic studies conducted. This influence is nicely seen in the crystal of the binary species, Zn[S_2_N(CH_2_CH_2_OH)_2_]_2_, where the dimeric aggregate, mediated by Zn—S bridges, self-assembles into a three-dimensional architecture based on hydrogen bonding (Benson *et al.*, 2007 ▸). Studies have shown that binuclear aggregates with 4,4′-bi­pyridine (bipy) bridges can be formed with these function­alized di­thio­carbamate ligands, consistent with the above, but extended arrays result, being stabilized *via* hydrogen bonding (Benson *et al.*, 2007 ▸), *e.g*. an open supra­molecular layer in the case of {Zn[S_2_CN(Me)CH_2_CH_2_OH]_2_}_2_(bipy), which allows for the construction of a doubly inter­penetrated architecture. When the bridge is pyrazine, the three-dimensional architecture is sustained by (hy­droxy)O—H⋯O(hy­droxy) hydrogen bonding exclusively (Jotani *et al.*, 2017 ▸). When the bridge is significantly longer, *e.g*. (3-pyrid­yl)CH_2_N(H)C(=O)C(=O)N(H)CH_2_(3-pyrid­yl), *i.e. L*H_2_, the dimeric {Zn[S_2_CN(Me)CH_2_CH_2_OH)_2_]_2_}_2_(*L*H_2_) aggregates are inter­woven into supra­molecular chains sustained by hydrogen bonding (Poplaukhin & Tiekink, 2010 ▸). In a continuation of these structural studies, herein the crystal and mol­ecular structures of two pyridine adducts are described, namely {Zn[S_2_CN(*R*)CH_2_CH_2_OH]_2_(pyridine)·pyridine} for *R* = Me (I) and Et (II).

## Structural commentary   

**Figure 1:**
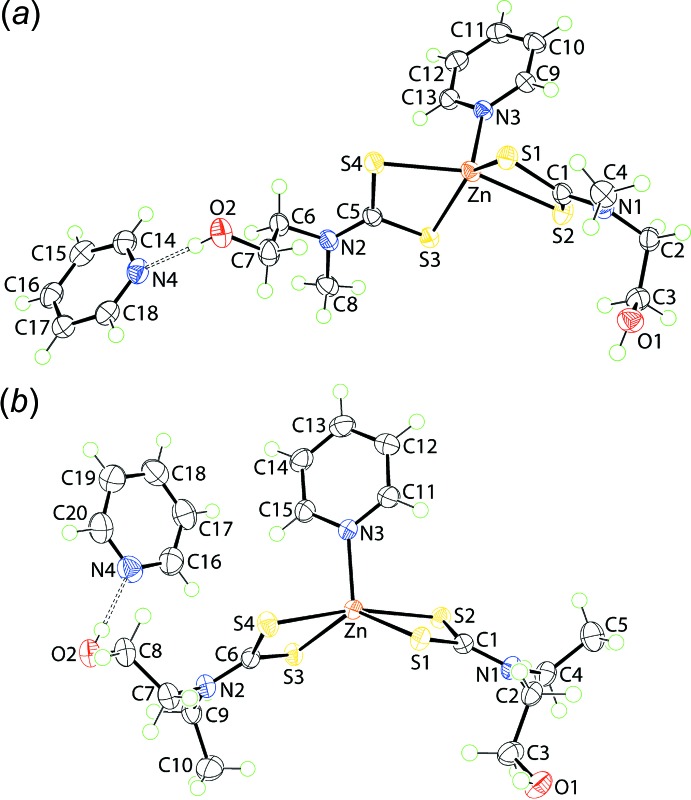
The mol­ecular structures of (*a*) (I) and (*b*) (II), showing the atom-labelling scheme and displacement ellipsoids at the 70% probability level.

The mol­ecular structures of {Zn[S_2_CN(*R*)CH_2_CH_2_OH]_2_(pyridine)·.pyridine}, for *R* = Me (I) and Et (II), are shown in Fig. 1 ▸, and selected geometric parameters are given in Table 1 ▸. In (I), the di­thio­carbamate ligands coordinate with non-symmetric Zn—S bond lengths which is conveniently qu­anti­fied by ΔZn—S = Zn—S_long_ - Zn—S_short_. For the S1-di­thio­carbamate ligand, ΔZn—S = 0.23 Å, but this decreases to 0.17 Å for the S3-ligand. From the data in Table 1 ▸, there is a tendency for the sulfur atoms forming the shorter Zn—S bonds to be involved in the longer C—S bonds. The pyridine-N atom occupies the fifth position in the five-coordinate zinc cation and forms N—Zn—S angles in the range 99.96 (4) to 111.99 (3)°. Further, the pyridine ring is almost orthogonal to the planes through each of the chelate rings, Table 1 ▸. The value of *τ*, which ranges from 0.0 to 1.0° for ideal square-pyramidal to trigonal-bipyramidal, respectively (Addison *et al.*, 1984 ▸), computes to 0.34, suggesting a distortion towards a square-pyramidal geometry. If this was the case, the basal plane comprises the four sulfur atoms (r.m.s. deviation = 0.1841 Å) and the zinc cation lies 0.6877 (3) Å out of the plane in the direction of the pyridine-N3 atom. The asymmetric unit of (I) is completed by a second pyridine mol­ecule that is connected to the (hy­droxy)O2—H atom *via* a hydrogen bond, Table 2 ▸.

To a first approximation the structure of (II) resembles that of (I). The values of ΔZn—S, at 0.27 and 0.19 Å for the S1- and S3-di­thio­carbamate ligands, respectively, are slightly greater than the equivalent values in (I), and this is also seen in the greater disparity in the associated C—S bond lengths, Table 1 ▸. The range of N—Zn—S bond angles is also broader, at 93.26 (5)–116.78 (5) Å and there is a disparity in the CS_2_/pyridine dihedral angles of 10.2°, *cf*. 1.1° for (I). The value of *τ* is 0.56, indicating a small tendency towards trigonal–bipyramidal, certainly when compared with the coordination geometry for (I). The widest angle subtended at the zinc cation is by the two less tightly held sulfur atoms, *i.e*. 166.375 (19)°. As for (I), distortions in the coordination geometry can be traced to the tight chelate angles, disparity in donor sets, bond lengths, *etc*. The solvent mol­ecule in (II) is also associated with the O2-hy­droxy group *via* a hydrogen bond.

**Figure 2:**
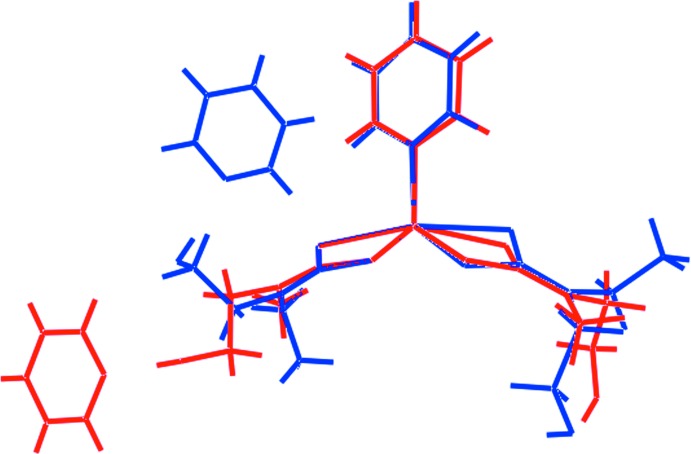
Overlay diagram of the asymmetric units of (I), red image, and (II). The mol­ecules have been overlapped so the more symmetrically coordinating di­thio­carbamate ligands are coincident.

Despite the relatively close agreement between the coord­ination geometries of the Zn[S_2_CN(*R*)CH_2_CH_2_OH]_2_ mol­ecules in (I) and (II), the overlay diagram shown in Fig. 2 ▸ emphasizes the differences in the relative orientations of the less symmetrically coordinating di­thio­carbamate ligands and the coordinating pyridine mol­ecules. More striking are the opposite orientations adopted by the hy­droxy groups of the more symmetrically coordinating di­thio­carbamate ligands and therefore, the pyridine mol­ecules to which they are connected. This impacts significantly upon the mol­ecular packing as described in the next Section.

## Supra­molecular features   

**Figure 3:**
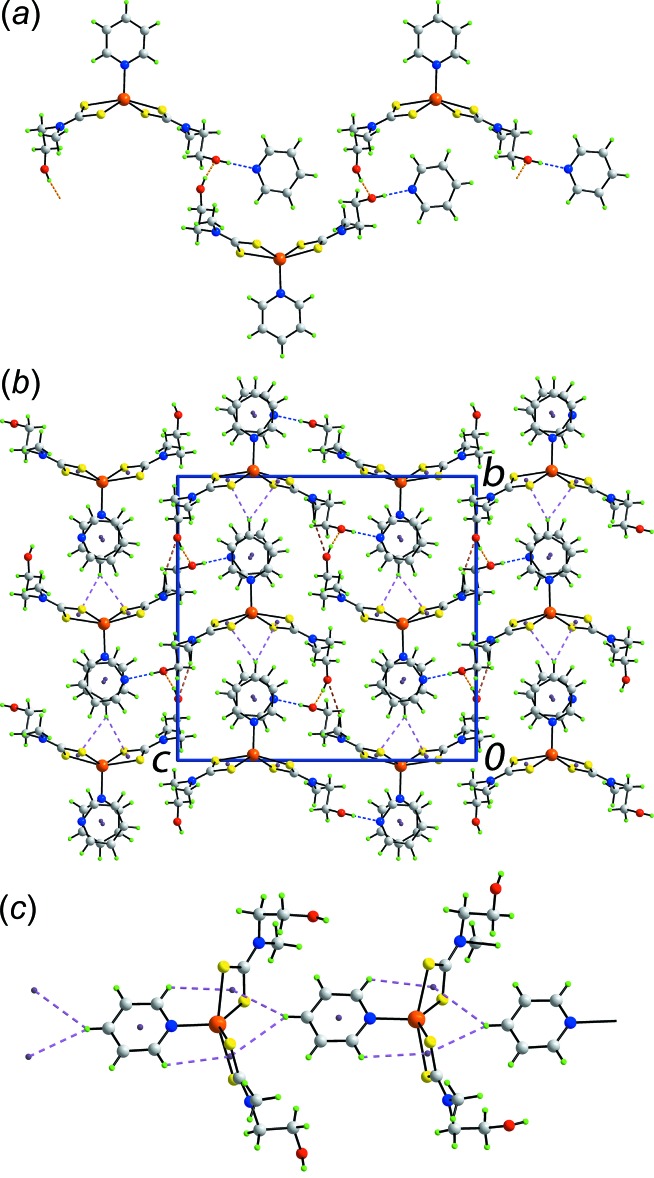
Mol­ecular packing in (I): (*a*) supra­molecular zigzag chain aligned along [10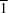] and sustained by O—H⋯O hydrogen bonding, with the solvent pyridine mol­ecules attached *via* O—H⋯N hydrogen bonding, (*b*) a view of the unit-cell contents in projection down the *a* axis and (*c*) supra­molecular chain along the *b* axis sustained by (pyridine)C—H⋯π(chelate ring) inter­actions. The O—H⋯O, O—H⋯N, C—H⋯O, π–π and C—H⋯π(chelate ring) inter­actions are shown as orange, blue, brown, purple and pink dashed lines, respectively.

The key geometric parameters characterizing the inter­molecular inter­actions operating in the crystals of (I) and (II) are collated in Tables 2 ▸ and 3 ▸, respectively. In the mol­ecular packing of (I), (hy­droxy)O—H⋯O(hy­droxy) hydrogen bonding between the two independent hy­droxy groups leads to a supra­molecular zigzag chain aligned along [10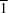] with the solvent pyridine mol­ecules associated with the chain *via* (hy­droxy)O—H⋯N(pyridine) hydrogen bonding, Fig. 3 ▸ *a*. While the hy­droxy-O2 atom is involved in two conventional hydrogen bonds, the hy­droxy-O1 atom is not. Rather, it participates in a (meth­yl)C—H⋯O(hy­droxy) inter­action, Table 2 ▸. Globally, mol­ecules stack along the *a* axis with solvent pyridine mol­ecules inter­spersed between coordinating pyridine mol­ecules to form columns connected by π(coordinating pyridine)—π(solvent pyridine) inter­actions so that each ring forms two contacts. The separations between the ring centroids are 3.4738 (10) and 3.4848 (10) Å for an angle of inclination = 0.28 (7)°; symmetry operations: 2 − *x*, 1 − *y*, −*z* and 1 − *x*, 1 − *y*, −*z*. Further connections between the constituent mol­ecules are of the type (solvent pyridine)C—H⋯π(chelate ring). As seen from Fig. 3 ▸ *a*, the solvent pyridine mol­ecules are located in a position proximate to the chelate rings enabling such inter­actions to form. A view of the unit-cell contents is shown in Fig. 3 ▸ *b*.

The result of the aforementioned (solvent pyridine)C—H⋯π(chelate ring) inter­actions is a supra­molecular chain aligned along the *b* axis as shown in Fig. 3 ▸ *c*. Such inter­actions are well known in the supra­molecular chemistry of metal 1,1-di­thiol­ates in general and di­thio­carbamates in particular owing to significant delocalization of π-electron density over the chelate rings relative to other 1,1-di­thiol­ate ligands such as those cited above (Tiekink & Zukerman-Schpector, 2011 ▸; Tiekink, 2017 ▸). The unusual feature in the present case is that the (pyridine)C—H hydrogen bond forming the inter­action is bifurcated (Tan *et al.*, 2016 ▸), Table 3 ▸. Finally, for completeness, it is noted that analogous but intra­molecular (coordinating pyridine)C—H⋯π(chelate ring) inter­actions also occur, Fig. 3 ▸ *c*, with (pyridine)C—H⋯ring centroid separations of 2 x 2.90 Å and C—H⋯ring centroid angles of 113°. These inter­actions might account for the symmetric disposition of the coordinating pyridine mol­ecule with respect to the ZnS_4_ arrangement.

**Figure 4:**
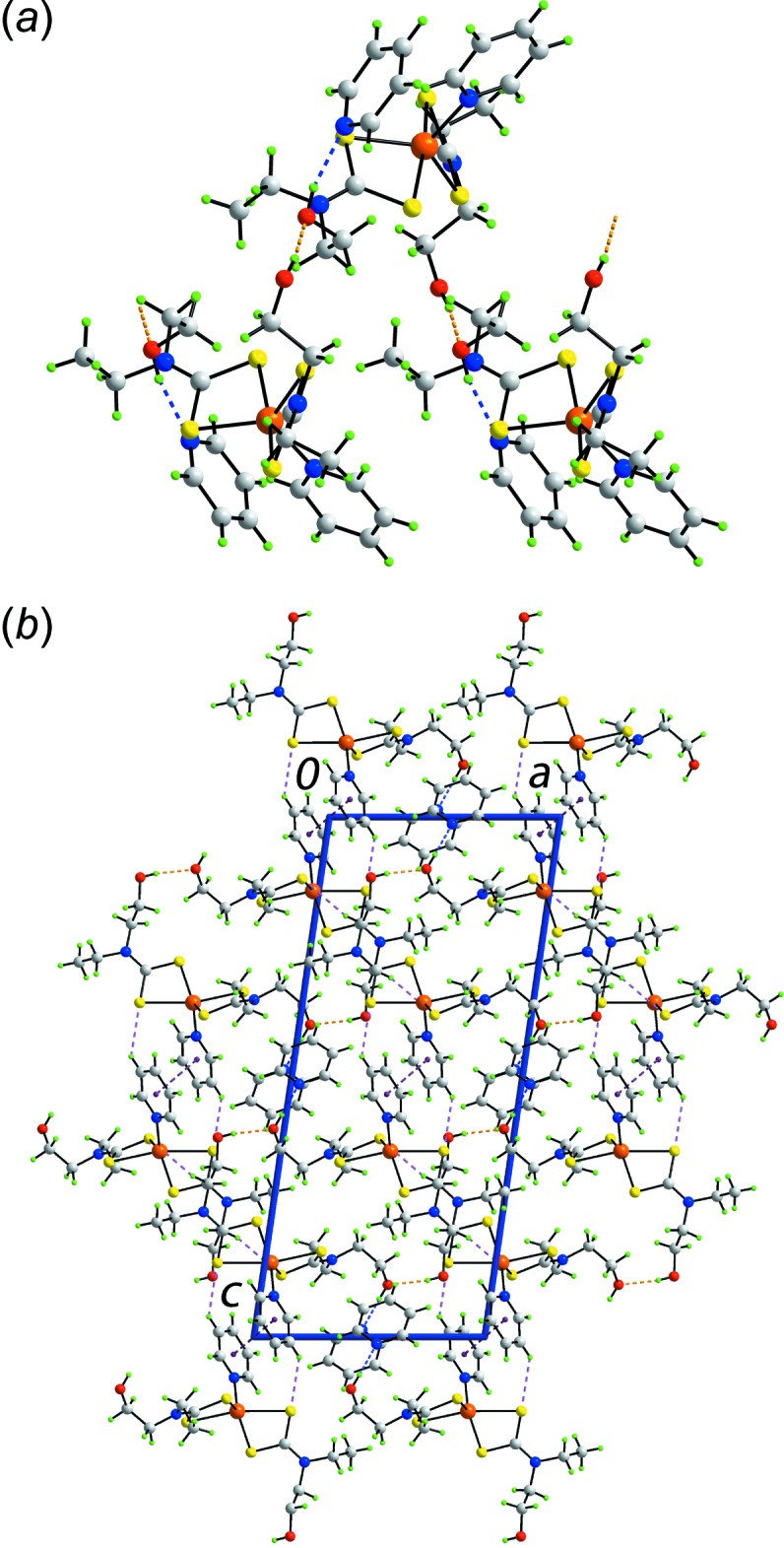
Mol­ecular packing in (II): (*a*) supra­molecular helical chain aligned along [010] and sustained by O—H⋯O hydrogen bonding, with the solvent pyridine mol­ecules attached *via* O—H⋯N hydrogen bonding, and (*b*) a view of the unit-cell contents in projection down the *a* axis. The O—H⋯S, O—H⋯N, C—H⋯S and π–π inter­actions are shown as orange, blue, pink and purple dashed lines, respectively.

In the crystal of (II), (hy­droxy)O—H⋯O(hy­droxy) hydrogen bonds between the two independent hy­droxy groups are also formed but, in this case, leading to a supra­molecular helical chain aligned along [010] and again with the solvent pyridine mol­ecules associated *via* (hy­droxy)O—H⋯N(pyridine) hydrogen bonding, Fig. 4 ▸ *a*. Connections between the chains are of the type (coordinating pyridine)- and (methyl­ene)C—H⋯S inter­actions as well as weak π–π contacts between centrosymmetrically related coordinating pyridine mol­ecules [inter-centroid separation = 3.9815 (14) Å for symmetry operation 1 − *x*, 1 − *y*, 1 − *z*]. A view of the unit-cell contents is shown in Fig. 4 ▸ *b*. As for (I), it is noted that intra­molecular coordinating (pyridine)C—H⋯π(chelate ring) inter­actions occur [(pyridine)C—H⋯ring centroid separations are 2.93 and 2.90 Å, and C—H⋯ring centroid angles are 110 and 112°].

## Database survey   

As a result of encouraging biological activities, *e.g*. as anti-cancer agents (Cvek *et al.*, 2008 ▸; Tan *et al.*, 2015 ▸) and for applications in tropical diseases (Manar *et al.*, 2017 ▸), as well as their utility as single-source precursors for the deposition of ZnS nanomaterials (Hrubaru *et al.*, 2016 ▸; Manar *et al.*, 2017 ▸), zinc di­thio­carbamates continue to be well studied. The compounds are generally binuclear as there are equal numbers of chelating and tridentate, *μ*_2_-bridging ligands, leading to distorted square-pyramidal coordination spheres (Tiekink, 2003 ▸). The structures of the precursor mol­ecules are readily disrupted by the addition of small donor mol­ecules such as in the present report with pyridine. Indeed, one of the first pyridine adducts of a zinc di­thio­carbamate to be described was that of Zn(S_2_CNEt_2_)_2_(pyridine), which was motivated by the desire to destroy the binuclear structure observed for the binary di­thio­carbamate compound to form a lighter (*i.e*. lower mol­ecular weight) species to facilitate chemical vapour deposition studies (Malik *et al.*, 1999 ▸). A search of the Cambridge Structural Database (Groom *et al.*, 2016 ▸) reveals over 25 ‘hits’ for related Zn(S_2_CN*RR*’)_2_(pyridine) species. More sophisticated monodentate nitro­gen-donor adducts are also known, such as substituted pyridines, *e.g*. 3-hy­droxy­pyridine (Jotani *et al.*, 2016 ▸), and non-aromatic donors such as piperidine (Zaeva *et al.*, 2011 ▸) and urotropine (hexa­methyl­ene­tetra­mine; Câmpian *et al.*, 2016 ▸). All of the adducts reveal similar mononuclear structures with NS_4_ coordination geometries, similar to those described above for (I) and (II). Finally, it is inter­esting to note that the aforementioned Zn(S_2_CNEt_2_)_2_(pyridine) adduct has also been characterized as a mono-pyridine solvate (Ivanov *et al.*, 1998 ▸), indicating that hydrogen bonding of the type observed in (I) and (II) is not a prerequisite for incorporation of solvent pyridine in the crystal.

## Synthesis and crystallization   

The Zn[S_2_CN(*R*)CH_2_CH_2_OH]_2_, *R* = Me and Et, precursors were prepared as per established procedures (Benson *et al.*, 2007 ▸). Crystals were of (I) were prepared in the following manner. In a typical experiment, Zn[S_2_CN(*R*)CH_2_CH_2_OH]_2_, *R* = Me and Et (50 mg), was dissolved in pyridine (10 ml) and carefully layered with hexa­nes (10 ml). Crystals were harvested directly from solution and mounted immediately onto the diffractometer to avoid loss of pyridine.

## Refinement   

Crystal data, data collection and structure refinement details are summarized in Table 4 ▸. For each of (I) and (II), carbon-bound H atoms were placed in calculated positions (C—H = 0.95–0.99 Å) and were included in the refinement in the riding model approximation, with *U*_iso_(H) set to 1.2–1.5*U*_eq_(C). The O-bound H atoms were located in difference-Fourier maps but were refined with a distance restraint of O—H = 0.84±0.01 Å, and with *U*_iso_(H) set to 1.5*U*_eq_(O). For (I), the maximum and minimum residual electron density peaks of 0.70 and 1.48 e Å^−3^, respectively, were located 1.03 and 1.02 Å from the Zn atom. For (II), the maximum and minimum residual electron density peaks of 0.92 and 1.61 e Å^−3^, respectively, were located 1.02 and 0.61 Å from the Zn atom.

## Supplementary Material

Crystal structure: contains datablock(s) I, II, global. DOI: 10.1107/S2056989017010568/wm5406sup1.cif

Structure factors: contains datablock(s) I. DOI: 10.1107/S2056989017010568/wm5406Isup2.hkl

Structure factors: contains datablock(s) II. DOI: 10.1107/S2056989017010568/wm5406IIsup3.hkl

CCDC references: 1562767, 1562766

Additional supporting information:  crystallographic information; 3D view; checkCIF report

## Figures and Tables

**Table 1:** Geometric data (Å, °) for (I) and (II)

Parameter	(I); *n* = 5	(II); *n* = 6
Zn—S1	2.3618 (5)	2.3414 (6)
Zn—S2	2.5902 (5)	2.6140 (6)
Zn—S3	2.3678 (5)	2.3666 (6)
Zn—S4	2.5436 (5)	2.5627 (6)
Zn—N3	2.0504 (13)	2.0611 (16)
C1—S1, S2	1.7331 (15), 1.7176 (15)	1.7357 (18), 1.7168 (19)
C(*n*)—S3, S4	1.7309 (15), 1.7171 (15)	1.7388 (18), 1.7195 (19)
S1—Zn—S2	73.012 (16)	72.621 (17)
S3—Zn—S4	73.765 (16)	73.534 (16)
S1—Zn—S3	136.711 (17)	132.86 (2)
S1—Zn—S4	98.906 (17)	98.846 (19)
S2—Zn—S3	97.326 (17)	104.146 (17)
S2—Zn—S4	157.363 (16)	166.375 (19)
S1—Zn—N3	111.99 (3)	116.78 (5)
S2—Zn—N3	99.96 (4)	93.26 (5)
S3—Zn—N3	111.23 (3)	110.34 (5)
S4—Zn—N3	102.66 (4)	100.14 (5)
S1,S2,C1/S3,S4,*C*(*n*)	46.16 (2)	49.06 (5)
S1,S2,C1/pyrid­yl	83.78 (5)	78.21 (7)
S3,S4,*C*(*n*)/pyrid­yl	84.93 (4)	88.39 (5)

**Table 2:** Hydrogen-bond geometry (Å, °) for (I) *Cg*1 and *Cg*2 are the centroids of the Zn/S1/S2/C1 and Zn/S3/S4/C5 rings, respectively.

*D*—H⋯*A*	*D*—H	H⋯*A*	*D*⋯*A*	*D*—H⋯*A*
O1—H1*O*⋯O2^i^	0.84 (2)	1.88 (2)	2.7008 (18)	164 (2)
O2—H2*O*⋯N4	0.85 (2)	1.87 (2)	2.713 (2)	178 (2)
C8—H8*C*⋯O1^ii^	0.98	2.56	3.418 (2)	146
C11—H11⋯*Cg*1^iii^	0.95	2.87	3.7324 (18)	151
C11—H11⋯*Cg*2^iii^	0.95	2.98	3.7772 (18)	142

Symmetry codes: (i) 
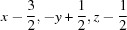
; (ii) 
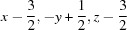
; (iii) 
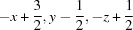
.

**Table 3:** Hydrogen-bond geometry (Å, °) for (II)

*D*—H⋯*A*	*D*—H	H⋯*A*	*D*⋯*A*	*D*—H⋯*A*
O1—H1*O*⋯O2^i^	0.84 (2)	1.99 (2)	2.817 (2)	167 (3)
O2—H2*O*⋯N4	0.84 (3)	1.93 (3)	2.753 (3)	167 (3)
C2—H2*B*⋯S3^ii^	0.99	2.84	3.773 (2)	157
C14—H14⋯S2^iii^	0.95	2.69	3.443 (2)	137

Symmetry codes: (i) 
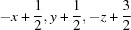
; (ii) 
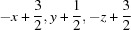
; (iii) 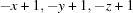.

**Table 4:** Experimental details

	(I)	(II)
Crystal data		
Chemical formula	[Zn(C_4_H_8_NOS_2_)_2_(C_5_H_5_N)]·C_5_H_5_N	[Zn(C_5_H_10_NOS_2_)_2_(C_5_H_5_N)]·C_5_H_5_N
*M*_r_	524.06	552.09
Crystal system, space group	Monoclinic, *P*2_1_/*n*	Monoclinic, *P*2_1_/*n*
Temperature (K)	98	98
*a*, *b*, *c* (Å)	6.9457 (9), 17.638 (2), 18.552 (3)	11.2961 (16), 8.6514 (12), 25.716 (4)
β (°)	91.619 (2)	98.265 (3)
*V* (Å^3^)	2271.9 (5)	2487.0 (6)
*Z*	4	4
Radiation type	Mo *K*α	Mo *K*α
μ (mm^−1^)	1.47	1.35
Crystal size (mm)	0.40 × 0.30 × 0.25	0.30 × 0.15 × 0.10

Data collection		
Diffractometer	AFC12K/SATURN724	AFC12K/SATURN724
Absorption correction	Multi-scan (*ABSCOR*; Higashi, 1995 ▸)	Multi-scan (*ABSCOR*; Higashi, 1995 ▸)
*T*_min_, *T*_max_	0.766, 1.000	0.823, 1.000
No. of measured, independent and observed [*I* > 2σ(*I*)] reflections	25102, 13197, 11207	38694, 15181, 12445
*R*_int_	0.031	0.044
(sin θ/λ)_max_ (Å^−1^)	0.918	0.920

Refinement		
*R*[*F* ^2^ > 2σ(*F* ^2^)], *wR*(*F* ^2^), *S*	0.056, 0.140, 1.09	0.067, 0.167, 1.12
No. of reflections	13197	15181
No. of parameters	270	288
No. of restraints	2	2
H-atom treatment	H atoms treated by a mixture of independent and constrained refinement	H atoms treated by a mixture of independent and constrained refinement
Δρ_max_, Δρ_min_ (e Å^−3^)	0.70, −1.48	0.92, −1.61

Computer programs: *CrystalClear* (Molecular Structure Corporation & Rigaku, 2005 ▸), *SHELXS* (Sheldrick, 2008 ▸), *SHELXL2014* (Sheldrick, 2015 ▸), *ORTEP-3 for Windows* (Farrugia, 2012 ▸), *QMol* (Gans & Shalloway, 2001 ▸), *DIAMOND* (Brandenburg, 2006 ▸) and *publCIF* (Westrip, 2010 ▸).

## supplementary crystallographic information

### Bis[N-(2-hydroxyethyl)-N-methyldithiocarbamato-κ2S,S'](pyridine)zinc(II) pyridine monosolvate (I) Crystal data

**Table:**

[Zn(C_4_H_8_NOS_2_)_2_(C_5_H_5_N)]·C_5_H_5_N	*F*(000) = 1088
*M**_r_* = 524.06	*D*_x_ = 1.532 Mg m^−^^3^
Monoclinic, *P*2_1_/*n*	Mo *K*α radiation, λ = 0.71069 Å
*a* = 6.9457 (9) Å	Cell parameters from 14301 reflections
*b* = 17.638 (2) Å	θ = 2.3–40.7°
*c* = 18.552 (3) Å	µ = 1.47 mm^−^^1^
β = 91.619 (2)°	*T* = 98 K
*V* = 2271.9 (5) Å^3^	Prism, colourless
*Z* = 4	0.40 × 0.30 × 0.25 mm

### Bis[N-(2-hydroxyethyl)-N-methyldithiocarbamato-κ2S,S'](pyridine)zinc(II) pyridine monosolvate (I) Data collection

**Table:**

AFC12K/SATURN724 diffractometer	13197 independent reflections
Radiation source: fine-focus sealed tube	11207 reflections with *I* > 2σ(*I*)
Graphite monochromator	*R*_int_ = 0.031
ω scans	θ_max_ = 40.7°, θ_min_ = 2.3°
Absorption correction: multi-scan (ABSCOR; Higashi, 1995)	*h* = −12→6
*T*_min_ = 0.766, *T*_max_ = 1.000	*k* = −32→24
25102 measured reflections	*l* = −24→33

### Bis[N-(2-hydroxyethyl)-N-methyldithiocarbamato-κ2S,S'](pyridine)zinc(II) pyridine monosolvate (I) Refinement

**Table:**

Refinement on *F*^2^	2 restraints
Least-squares matrix: full	Hydrogen site location: inferred from neighbouring sites
*R*[*F*^2^ > 2σ(*F*^2^)] = 0.056	H atoms treated by a mixture of independent and constrained refinement
*wR*(*F*^2^) = 0.140	*w* = 1/[σ^2^(*F*_o_^2^) + (0.053*P*)^2^ + 1.0614*P*] where *P* = (*F*_o_^2^ + 2*F*_c_^2^)/3
*S* = 1.09	(Δ/σ)_max_ = 0.001
13197 reflections	Δρ_max_ = 0.70 e Å^−^^3^
270 parameters	Δρ_min_ = −1.48 e Å^−^^3^

### Bis[N-(2-hydroxyethyl)-N-methyldithiocarbamato-κ2S,S'](pyridine)zinc(II) pyridine monosolvate (I) Special details

**Table:**

Geometry. All esds (except the esd in the dihedral angle between two l.s. planes) are estimated using the full covariance matrix. The cell esds are taken into account individually in the estimation of esds in distances, angles and torsion angles; correlations between esds in cell parameters are only used when they are defined by crystal symmetry. An approximate (isotropic) treatment of cell esds is used for estimating esds involving l.s. planes.

### Bis[N-(2-hydroxyethyl)-N-methyldithiocarbamato-κ2S,S'](pyridine)zinc(II) pyridine monosolvate (I) Fractional atomic coordinates and isotropic or equivalent isotropic displacement parameters (Å^2^)

**Table:**

	*x*	*y*	*z*	*U*_iso_*/*U*_eq_	
Zn	0.76451 (3)	0.48081 (2)	0.24723 (2)	0.01870 (5)	
S1	1.01537 (5)	0.53140 (2)	0.32115 (2)	0.02020 (7)	
S2	0.62546 (6)	0.50167 (2)	0.37379 (2)	0.02190 (7)	
S3	0.49806 (5)	0.52874 (2)	0.17907 (2)	0.01974 (7)	
S4	0.89209 (6)	0.51679 (2)	0.12469 (2)	0.02174 (7)	
O1	0.74031 (18)	0.71648 (7)	0.50152 (7)	0.0249 (2)	
H1O	0.673 (3)	0.7534 (10)	0.4870 (14)	0.037*	
O2	0.96838 (19)	0.68555 (7)	−0.04784 (7)	0.0271 (2)	
H2O	0.912 (3)	0.6949 (15)	−0.0879 (8)	0.041*	
N1	0.59677 (19)	0.57633 (7)	0.04818 (7)	0.0197 (2)	
N2	0.90043 (19)	0.57235 (7)	0.45207 (7)	0.0195 (2)	
N3	0.77129 (17)	0.36463 (7)	0.24459 (6)	0.01762 (19)	
N4	0.7983 (2)	0.71567 (9)	−0.17816 (8)	0.0242 (2)	
C1	0.8516 (2)	0.53850 (8)	0.38962 (7)	0.0178 (2)	
C2	0.7607 (2)	0.58086 (8)	0.50946 (8)	0.0212 (2)	
H2A	0.8312	0.5863	0.5563	0.025*	
H2B	0.6813	0.5344	0.5117	0.025*	
C3	0.6293 (2)	0.64871 (9)	0.49820 (9)	0.0235 (3)	
H3A	0.5617	0.6449	0.4506	0.028*	
H3B	0.5313	0.6496	0.5359	0.028*	
C4	1.0894 (2)	0.60795 (9)	0.46490 (9)	0.0240 (3)	
H4A	1.1826	0.5859	0.4322	0.036*	
H4B	1.1326	0.5991	0.5149	0.036*	
H4C	1.0792	0.6626	0.4561	0.036*	
C5	0.6568 (2)	0.54396 (8)	0.11027 (7)	0.0177 (2)	
C6	0.7319 (2)	0.59353 (8)	−0.00895 (8)	0.0208 (2)	
H6A	0.6629	0.5923	−0.0564	0.025*	
H6B	0.8343	0.5545	−0.0092	0.025*	
C7	0.8224 (3)	0.67163 (9)	0.00288 (9)	0.0262 (3)	
H7A	0.7215	0.7111	−0.0021	0.031*	
H7B	0.8790	0.6747	0.0524	0.031*	
C8	0.3984 (2)	0.60330 (9)	0.03608 (9)	0.0239 (3)	
H8A	0.3140	0.5788	0.0705	0.036*	
H8B	0.3547	0.5907	−0.0132	0.036*	
H8C	0.3941	0.6584	0.0428	0.036*	
C9	0.8304 (2)	0.32452 (8)	0.30251 (8)	0.0195 (2)	
H9	0.8710	0.3509	0.3449	0.023*	
C10	0.8342 (2)	0.24592 (9)	0.30270 (9)	0.0230 (3)	
H10	0.8770	0.2189	0.3445	0.028*	
C11	0.7742 (2)	0.20738 (9)	0.24053 (10)	0.0244 (3)	
H11	0.7753	0.1535	0.2392	0.029*	
C12	0.7130 (2)	0.24853 (9)	0.18064 (9)	0.0227 (3)	
H12	0.6708	0.2235	0.1377	0.027*	
C13	0.7145 (2)	0.32700 (8)	0.18455 (8)	0.0200 (2)	
H13	0.6739	0.3553	0.1433	0.024*	
C14	0.7583 (2)	0.65504 (10)	−0.21958 (10)	0.0254 (3)	
H14	0.7787	0.6060	−0.1997	0.030*	
C15	0.6883 (2)	0.66082 (10)	−0.29017 (10)	0.0266 (3)	
H15	0.6625	0.6167	−0.3181	0.032*	
C16	0.6568 (2)	0.73237 (11)	−0.31896 (9)	0.0266 (3)	
H16	0.6091	0.7381	−0.3672	0.032*	
C17	0.6958 (2)	0.79563 (10)	−0.27655 (9)	0.0251 (3)	
H17	0.6742	0.8453	−0.2949	0.030*	
C18	0.7671 (2)	0.78459 (10)	−0.20678 (9)	0.0242 (3)	
H18	0.7952	0.8279	−0.1779	0.029*	

### Bis[N-(2-hydroxyethyl)-N-methyldithiocarbamato-κ2S,S'](pyridine)zinc(II) pyridine monosolvate (I) Atomic displacement parameters (Å^2^)

**Table:**

	*U*^11^	*U*^22^	*U*^33^	*U*^12^	*U*^13^	*U*^23^
Zn	0.02335 (9)	0.01525 (7)	0.01732 (8)	0.00012 (5)	−0.00244 (6)	0.00023 (5)
S1	0.02114 (15)	0.02096 (14)	0.01852 (14)	−0.00256 (11)	0.00097 (12)	−0.00106 (11)
S2	0.02227 (16)	0.02518 (16)	0.01831 (14)	−0.00572 (12)	0.00155 (12)	−0.00305 (12)
S3	0.02062 (15)	0.02014 (14)	0.01851 (14)	0.00136 (11)	0.00147 (12)	0.00278 (11)
S4	0.02038 (15)	0.02581 (16)	0.01907 (15)	0.00265 (12)	0.00130 (12)	0.00458 (11)
O1	0.0293 (6)	0.0186 (4)	0.0266 (5)	−0.0002 (4)	−0.0016 (4)	−0.0008 (4)
O2	0.0313 (6)	0.0283 (5)	0.0217 (5)	−0.0070 (4)	0.0008 (4)	0.0036 (4)
N1	0.0234 (5)	0.0184 (5)	0.0173 (5)	0.0002 (4)	−0.0007 (4)	0.0023 (4)
N2	0.0236 (5)	0.0177 (4)	0.0170 (5)	−0.0017 (4)	−0.0012 (4)	−0.0007 (4)
N3	0.0192 (5)	0.0162 (4)	0.0174 (5)	−0.0002 (3)	−0.0005 (4)	0.0006 (3)
N4	0.0208 (5)	0.0307 (6)	0.0210 (5)	−0.0014 (5)	0.0015 (4)	0.0012 (5)
C1	0.0218 (6)	0.0152 (5)	0.0163 (5)	−0.0018 (4)	−0.0009 (4)	0.0008 (4)
C2	0.0281 (7)	0.0193 (5)	0.0162 (5)	−0.0008 (5)	0.0003 (5)	−0.0002 (4)
C3	0.0271 (7)	0.0205 (6)	0.0228 (6)	0.0002 (5)	0.0011 (5)	−0.0023 (5)
C4	0.0257 (7)	0.0206 (6)	0.0255 (7)	−0.0040 (5)	−0.0050 (6)	−0.0011 (5)
C5	0.0211 (5)	0.0153 (5)	0.0168 (5)	0.0000 (4)	−0.0011 (4)	0.0000 (4)
C6	0.0269 (6)	0.0195 (5)	0.0159 (5)	−0.0015 (5)	0.0003 (5)	0.0008 (4)
C7	0.0373 (8)	0.0205 (6)	0.0211 (6)	−0.0052 (5)	0.0038 (6)	0.0005 (5)
C8	0.0245 (6)	0.0243 (6)	0.0226 (6)	0.0025 (5)	−0.0044 (5)	0.0023 (5)
C9	0.0205 (6)	0.0186 (5)	0.0193 (5)	0.0001 (4)	−0.0010 (4)	0.0027 (4)
C10	0.0222 (6)	0.0195 (5)	0.0273 (7)	0.0013 (5)	0.0020 (5)	0.0065 (5)
C11	0.0232 (6)	0.0166 (5)	0.0336 (8)	−0.0009 (4)	0.0036 (6)	0.0008 (5)
C12	0.0226 (6)	0.0197 (6)	0.0258 (7)	−0.0018 (5)	0.0012 (5)	−0.0048 (5)
C13	0.0215 (6)	0.0192 (5)	0.0192 (5)	−0.0004 (4)	−0.0005 (5)	−0.0009 (4)
C14	0.0217 (6)	0.0255 (7)	0.0292 (7)	−0.0020 (5)	0.0053 (5)	0.0015 (5)
C15	0.0222 (6)	0.0298 (7)	0.0280 (7)	−0.0044 (5)	0.0031 (6)	−0.0072 (6)
C16	0.0215 (6)	0.0376 (8)	0.0205 (6)	0.0012 (6)	0.0002 (5)	−0.0023 (6)
C17	0.0236 (6)	0.0277 (7)	0.0240 (6)	0.0036 (5)	0.0031 (5)	0.0020 (5)
C18	0.0213 (6)	0.0274 (7)	0.0241 (6)	−0.0008 (5)	0.0024 (5)	−0.0029 (5)

### Bis[N-(2-hydroxyethyl)-N-methyldithiocarbamato-κ2S,S'](pyridine)zinc(II) pyridine monosolvate (I) Geometric parameters (Å, º)

**Table:**

Zn—N3	2.0504 (13)	C4—H4B	0.9800
Zn—S1	2.3618 (5)	C4—H4C	0.9800
Zn—S3	2.3678 (5)	C6—C7	1.528 (2)
Zn—S4	2.5436 (5)	C6—H6A	0.9900
Zn—S2	2.5902 (5)	C6—H6B	0.9900
S1—C1	1.7331 (15)	C7—H7A	0.9900
S2—C1	1.7176 (15)	C7—H7B	0.9900
S3—C5	1.7309 (15)	C8—H8A	0.9800
S4—C5	1.7171 (15)	C8—H8B	0.9800
O1—C3	1.423 (2)	C8—H8C	0.9800
O1—H1O	0.840 (9)	C9—C10	1.387 (2)
O2—C7	1.424 (2)	C9—H9	0.9500
O2—H2O	0.847 (9)	C10—C11	1.392 (2)
N1—C5	1.3412 (19)	C10—H10	0.9500
N1—C6	1.467 (2)	C11—C12	1.384 (2)
N1—C8	1.469 (2)	C11—H11	0.9500
N2—C1	1.3385 (18)	C12—C13	1.386 (2)
N2—C2	1.469 (2)	C12—H12	0.9500
N2—C4	1.469 (2)	C13—H13	0.9500
N3—C9	1.3410 (18)	C14—C15	1.388 (3)
N3—C13	1.3462 (19)	C14—H14	0.9500
N4—C14	1.341 (2)	C15—C16	1.385 (3)
N4—C18	1.342 (2)	C15—H15	0.9500
C2—C3	1.516 (2)	C16—C17	1.388 (2)
C2—H2A	0.9900	C16—H16	0.9500
C2—H2B	0.9900	C17—C18	1.386 (3)
C3—H3A	0.9900	C17—H17	0.9500
C3—H3B	0.9900	C18—H18	0.9500
C4—H4A	0.9800		
			
N3—Zn—S1	111.99 (3)	S4—C5—S3	117.71 (8)
N3—Zn—S3	111.23 (3)	N1—C6—C7	110.61 (12)
S1—Zn—S3	136.711 (17)	N1—C6—H6A	109.5
N3—Zn—S4	102.66 (4)	C7—C6—H6A	109.5
S1—Zn—S4	98.906 (17)	N1—C6—H6B	109.5
S3—Zn—S4	73.765 (16)	C7—C6—H6B	109.5
N3—Zn—S2	99.96 (4)	H6A—C6—H6B	108.1
S1—Zn—S2	73.012 (16)	O2—C7—C6	111.01 (13)
S3—Zn—S2	97.326 (17)	O2—C7—H7A	109.4
S4—Zn—S2	157.363 (16)	C6—C7—H7A	109.4
C1—S1—Zn	87.95 (5)	O2—C7—H7B	109.4
C1—S2—Zn	81.14 (5)	C6—C7—H7B	109.4
C5—S3—Zn	86.84 (5)	H7A—C7—H7B	108.0
C5—S4—Zn	81.65 (5)	N1—C8—H8A	109.5
C3—O1—H1O	110.0 (18)	N1—C8—H8B	109.5
C7—O2—H2O	107.1 (19)	H8A—C8—H8B	109.5
C5—N1—C6	121.26 (13)	N1—C8—H8C	109.5
C5—N1—C8	122.44 (13)	H8A—C8—H8C	109.5
C6—N1—C8	116.04 (12)	H8B—C8—H8C	109.5
C1—N2—C2	121.13 (13)	N3—C9—C10	122.33 (14)
C1—N2—C4	122.30 (13)	N3—C9—H9	118.8
C2—N2—C4	116.41 (12)	C10—C9—H9	118.8
C9—N3—C13	118.61 (13)	C9—C10—C11	118.73 (14)
C9—N3—Zn	120.98 (10)	C9—C10—H10	120.6
C13—N3—Zn	120.40 (10)	C11—C10—H10	120.6
C14—N4—C18	117.84 (15)	C12—C11—C10	119.15 (15)
N2—C1—S2	121.64 (11)	C12—C11—H11	120.4
N2—C1—S1	120.75 (11)	C10—C11—H11	120.4
S2—C1—S1	117.60 (8)	C11—C12—C13	118.69 (15)
N2—C2—C3	112.86 (12)	C11—C12—H12	120.7
N2—C2—H2A	109.0	C13—C12—H12	120.7
C3—C2—H2A	109.0	N3—C13—C12	122.49 (14)
N2—C2—H2B	109.0	N3—C13—H13	118.8
C3—C2—H2B	109.0	C12—C13—H13	118.8
H2A—C2—H2B	107.8	N4—C14—C15	122.92 (16)
O1—C3—C2	109.50 (13)	N4—C14—H14	118.5
O1—C3—H3A	109.8	C15—C14—H14	118.5
C2—C3—H3A	109.8	C16—C15—C14	118.56 (15)
O1—C3—H3B	109.8	C16—C15—H15	120.7
C2—C3—H3B	109.8	C14—C15—H15	120.7
H3A—C3—H3B	108.2	C15—C16—C17	119.20 (16)
N2—C4—H4A	109.5	C15—C16—H16	120.4
N2—C4—H4B	109.5	C17—C16—H16	120.4
H4A—C4—H4B	109.5	C18—C17—C16	118.38 (15)
N2—C4—H4C	109.5	C18—C17—H17	120.8
H4A—C4—H4C	109.5	C16—C17—H17	120.8
H4B—C4—H4C	109.5	N4—C18—C17	123.10 (15)
N1—C5—S4	121.59 (11)	N4—C18—H18	118.5
N1—C5—S3	120.70 (11)	C17—C18—H18	118.5
			
C2—N2—C1—S2	−1.26 (19)	Zn—S3—C5—S4	1.93 (7)
C4—N2—C1—S2	−176.42 (11)	C5—N1—C6—C7	−87.22 (17)
C2—N2—C1—S1	177.95 (10)	C8—N1—C6—C7	87.01 (16)
C4—N2—C1—S1	2.79 (19)	N1—C6—C7—O2	173.90 (13)
Zn—S2—C1—N2	174.33 (12)	C13—N3—C9—C10	−0.2 (2)
Zn—S2—C1—S1	−4.90 (7)	Zn—N3—C9—C10	179.15 (11)
Zn—S1—C1—N2	−173.92 (12)	N3—C9—C10—C11	−0.1 (2)
Zn—S1—C1—S2	5.32 (8)	C9—C10—C11—C12	0.1 (2)
C1—N2—C2—C3	−82.38 (17)	C10—C11—C12—C13	0.3 (2)
C4—N2—C2—C3	93.05 (16)	C9—N3—C13—C12	0.7 (2)
N2—C2—C3—O1	−64.00 (16)	Zn—N3—C13—C12	−178.73 (12)
C6—N1—C5—S4	−3.14 (19)	C11—C12—C13—N3	−0.7 (2)
C8—N1—C5—S4	−177.00 (11)	C18—N4—C14—C15	0.6 (2)
C6—N1—C5—S3	176.46 (10)	N4—C14—C15—C16	−0.6 (3)
C8—N1—C5—S3	2.6 (2)	C14—C15—C16—C17	−0.1 (2)
Zn—S4—C5—N1	177.80 (12)	C15—C16—C17—C18	0.7 (2)
Zn—S4—C5—S3	−1.81 (7)	C14—N4—C18—C17	0.1 (2)
Zn—S3—C5—N1	−177.69 (12)	C16—C17—C18—N4	−0.7 (3)

### Bis[N-(2-hydroxyethyl)-N-methyldithiocarbamato-κ2S,S'](pyridine)zinc(II) pyridine monosolvate (I) Hydrogen-bond geometry (Å, º)

*Cg*1 and *Cg*2 are the centroids of the Zn/S1/S2/C1 and Zn/S3/S4/C5 rings, respectively.

**Table:**

*D*—H···*A*	*D*—H	H···*A*	*D*···*A*	*D*—H···*A*
O1—H1*O*···O2^i^	0.84 (2)	1.88 (2)	2.7008 (18)	164 (2)
O2—H2*O*···N4	0.85 (2)	1.87 (2)	2.713 (2)	178 (2)
C8—H8*C*···O1^ii^	0.98	2.56	3.418 (2)	146
C11—H11···*Cg*1^iii^	0.95	2.87	3.7324 (18)	151
C11—H11···*Cg*2^iii^	0.95	2.98	3.7772 (18)	142

Symmetry codes: (i) *x*−3/2, −*y*+1/2, *z*−1/2; (ii) *x*−3/2, −*y*+1/2, *z*−3/2; (iii) −*x*+3/2, *y*−1/2, −*z*+1/2.

### Bis[N-ethyl-N-(2-hydroxyethyl)dithiocarbamato-κ2S,S'](pyridine)zinc(II) pyridine monosolvate (II) Crystal data

**Table:**

[Zn(C_5_H_10_NOS_2_)_2_(C_5_H_5_N)]·C_5_H_5_N	*F*(000) = 1152
*M**_r_* = 552.09	*D*_x_ = 1.474 Mg m^−^^3^
Monoclinic, *P*2_1_/*n*	Mo *K*α radiation, λ = 0.71069 Å
*a* = 11.2961 (16) Å	Cell parameters from 12656 reflections
*b* = 8.6514 (12) Å	θ = 2.4–40.8°
*c* = 25.716 (4) Å	µ = 1.35 mm^−^^1^
β = 98.265 (3)°	*T* = 98 K
*V* = 2487.0 (6) Å^3^	Prism, colourless
*Z* = 4	0.30 × 0.15 × 0.10 mm

### Bis[N-ethyl-N-(2-hydroxyethyl)dithiocarbamato-κ2S,S'](pyridine)zinc(II) pyridine monosolvate (II) Data collection

**Table:**

AFC12K/SATURN724 diffractometer	15181 independent reflections
Radiation source: fine-focus sealed tube	12445 reflections with *I* > 2σ(*I*)
Graphite monochromator	*R*_int_ = 0.044
ω scans	θ_max_ = 40.8°, θ_min_ = 2.5°
Absorption correction: multi-scan (ABSCOR; Higashi, 1995)	*h* = −20→20
*T*_min_ = 0.823, *T*_max_ = 1.000	*k* = −14→15
38694 measured reflections	*l* = −44→34

### Bis[N-ethyl-N-(2-hydroxyethyl)dithiocarbamato-κ2S,S'](pyridine)zinc(II) pyridine monosolvate (II) Refinement

**Table:**

Refinement on *F*^2^	2 restraints
Least-squares matrix: full	Hydrogen site location: inferred from neighbouring sites
*R*[*F*^2^ > 2σ(*F*^2^)] = 0.067	H atoms treated by a mixture of independent and constrained refinement
*wR*(*F*^2^) = 0.167	*w* = 1/[σ^2^(*F*_o_^2^) + (0.0606*P*)^2^ + 2.0316*P*] where *P* = (*F*_o_^2^ + 2*F*_c_^2^)/3
*S* = 1.12	(Δ/σ)_max_ = 0.003
15181 reflections	Δρ_max_ = 0.92 e Å^−^^3^
288 parameters	Δρ_min_ = −1.61 e Å^−^^3^

### Bis[N-ethyl-N-(2-hydroxyethyl)dithiocarbamato-κ2S,S'](pyridine)zinc(II) pyridine monosolvate (II) Special details

**Table:**

Geometry. All esds (except the esd in the dihedral angle between two l.s. planes) are estimated using the full covariance matrix. The cell esds are taken into account individually in the estimation of esds in distances, angles and torsion angles; correlations between esds in cell parameters are only used when they are defined by crystal symmetry. An approximate (isotropic) treatment of cell esds is used for estimating esds involving l.s. planes.

### Bis[N-ethyl-N-(2-hydroxyethyl)dithiocarbamato-κ2S,S'](pyridine)zinc(II) pyridine monosolvate (II) Fractional atomic coordinates and isotropic or equivalent isotropic displacement parameters (Å^2^)

**Table:**

	*x*	*y*	*z*	*U*_iso_*/*U*_eq_	
Zn	0.47427 (2)	0.44028 (3)	0.64271 (2)	0.01851 (5)	
S1	0.55997 (4)	0.55454 (6)	0.72177 (2)	0.01876 (8)	
S2	0.70555 (4)	0.44837 (5)	0.64257 (2)	0.01879 (8)	
S3	0.42049 (4)	0.18271 (5)	0.61898 (2)	0.01930 (8)	
S4	0.26154 (4)	0.40105 (6)	0.66400 (2)	0.02107 (9)	
O1	0.78229 (14)	0.4876 (2)	0.88352 (6)	0.0266 (3)	
H1O	0.7169 (18)	0.521 (4)	0.8910 (14)	0.040*	
O2	−0.04706 (14)	0.05941 (19)	0.60039 (7)	0.0250 (3)	
H2O	−0.019 (3)	0.082 (4)	0.5728 (9)	0.038*	
N1	0.79784 (14)	0.52998 (19)	0.74066 (7)	0.0192 (3)	
N2	0.21137 (14)	0.10140 (19)	0.64833 (7)	0.0191 (3)	
N3	0.43968 (14)	0.58368 (19)	0.57831 (6)	0.0187 (2)	
N4	0.03600 (19)	0.1826 (3)	0.51379 (8)	0.0305 (4)	
C1	0.70026 (15)	0.5128 (2)	0.70533 (7)	0.0174 (3)	
C2	0.79109 (17)	0.5804 (2)	0.79481 (7)	0.0205 (3)	
H2A	0.7252	0.6560	0.7946	0.025*	
H2B	0.8667	0.6323	0.8094	0.025*	
C3	0.7695 (2)	0.4432 (2)	0.82968 (8)	0.0254 (4)	
H3A	0.6879	0.4021	0.8188	0.030*	
H3B	0.8274	0.3600	0.8252	0.030*	
C4	0.91884 (16)	0.4924 (2)	0.72956 (8)	0.0221 (3)	
H4A	0.9121	0.4266	0.6978	0.027*	
H4B	0.9610	0.4319	0.7593	0.027*	
C5	0.9930 (2)	0.6340 (3)	0.72107 (10)	0.0293 (4)	
H5A	0.9530	0.6931	0.6911	0.044*	
H5B	1.0723	0.6016	0.7140	0.044*	
H5C	1.0015	0.6989	0.7526	0.044*	
C6	0.28789 (15)	0.2160 (2)	0.64413 (7)	0.0177 (3)	
C7	0.10530 (17)	0.1225 (2)	0.67473 (8)	0.0225 (3)	
H7A	0.1238	0.2000	0.7031	0.027*	
H7B	0.0869	0.0236	0.6912	0.027*	
C8	−0.00393 (18)	0.1750 (2)	0.63788 (9)	0.0245 (3)	
H8A	−0.0681	0.2023	0.6587	0.029*	
H8B	0.0163	0.2691	0.6191	0.029*	
C9	0.23077 (18)	−0.0579 (2)	0.63059 (8)	0.0218 (3)	
H9A	0.2790	−0.0544	0.6014	0.026*	
H9B	0.1525	−0.1050	0.6171	0.026*	
C10	0.2944 (2)	−0.1585 (3)	0.67444 (11)	0.0345 (5)	
H10A	0.3766	−0.1216	0.6842	0.052*	
H10B	0.2957	−0.2658	0.6623	0.052*	
H10C	0.2519	−0.1529	0.7050	0.052*	
C11	0.4984 (2)	0.7171 (2)	0.57605 (8)	0.0261 (4)	
H11	0.5525	0.7493	0.6058	0.031*	
C12	0.4829 (2)	0.8101 (3)	0.53160 (10)	0.0308 (4)	
H12	0.5255	0.9046	0.5311	0.037*	
C13	0.4043 (2)	0.7634 (3)	0.48801 (9)	0.0264 (4)	
H13	0.3918	0.8253	0.4572	0.032*	
C14	0.34453 (19)	0.6245 (3)	0.49038 (8)	0.0247 (3)	
H14	0.2910	0.5887	0.4610	0.030*	
C15	0.36386 (18)	0.5381 (2)	0.53632 (8)	0.0223 (3)	
H15	0.3219	0.4435	0.5380	0.027*	
C16	0.1423 (2)	0.1930 (3)	0.49671 (11)	0.0329 (5)	
H16	0.2075	0.1355	0.5144	0.039*	
C17	0.1613 (2)	0.2840 (3)	0.45431 (11)	0.0331 (5)	
H17	0.2376	0.2875	0.4430	0.040*	
C18	0.0669 (3)	0.3698 (3)	0.42880 (10)	0.0333 (5)	
H18	0.0776	0.4342	0.3999	0.040*	
C19	−0.0426 (3)	0.3600 (3)	0.44606 (11)	0.0363 (5)	
H19	−0.1091	0.4175	0.4294	0.044*	
C20	−0.0536 (2)	0.2647 (3)	0.48810 (11)	0.0345 (5)	
H20	−0.1298	0.2571	0.4994	0.041*	

### Bis[N-ethyl-N-(2-hydroxyethyl)dithiocarbamato-κ2S,S'](pyridine)zinc(II) pyridine monosolvate (II) Atomic displacement parameters (Å^2^)

**Table:**

	*U*^11^	*U*^22^	*U*^33^	*U*^12^	*U*^13^	*U*^23^
Zn	0.01866 (9)	0.01800 (9)	0.01757 (10)	−0.00288 (6)	−0.00185 (7)	0.00161 (6)
S1	0.01561 (16)	0.02345 (19)	0.01692 (18)	−0.00013 (13)	0.00130 (13)	−0.00153 (14)
S2	0.01858 (17)	0.02160 (19)	0.01603 (18)	−0.00044 (13)	0.00187 (13)	−0.00093 (13)
S3	0.01541 (16)	0.01877 (17)	0.0240 (2)	−0.00086 (13)	0.00388 (14)	−0.00173 (14)
S4	0.01822 (17)	0.01765 (18)	0.0277 (2)	−0.00105 (13)	0.00452 (15)	−0.00333 (15)
O1	0.0226 (6)	0.0376 (8)	0.0186 (6)	0.0032 (6)	−0.0002 (5)	0.0005 (6)
O2	0.0206 (6)	0.0278 (7)	0.0269 (7)	−0.0051 (5)	0.0040 (5)	−0.0044 (5)
N1	0.0155 (5)	0.0211 (6)	0.0201 (7)	0.0018 (5)	−0.0001 (5)	−0.0021 (5)
N2	0.0168 (6)	0.0185 (6)	0.0223 (7)	−0.0018 (4)	0.0039 (5)	−0.0020 (5)
N3	0.0173 (6)	0.0200 (6)	0.0179 (6)	−0.0015 (5)	−0.0009 (5)	0.0008 (5)
N4	0.0324 (9)	0.0331 (10)	0.0265 (9)	−0.0055 (7)	0.0055 (7)	−0.0009 (7)
C1	0.0170 (6)	0.0171 (6)	0.0179 (7)	0.0001 (5)	0.0021 (5)	0.0005 (5)
C2	0.0203 (7)	0.0219 (7)	0.0182 (7)	−0.0001 (5)	−0.0016 (5)	−0.0037 (6)
C3	0.0305 (9)	0.0247 (9)	0.0195 (8)	0.0015 (7)	−0.0011 (7)	−0.0007 (6)
C4	0.0173 (7)	0.0228 (8)	0.0257 (8)	0.0030 (6)	0.0010 (6)	−0.0034 (6)
C5	0.0244 (9)	0.0304 (10)	0.0343 (11)	−0.0031 (7)	0.0081 (8)	−0.0063 (8)
C6	0.0152 (6)	0.0183 (6)	0.0191 (7)	0.0004 (5)	0.0006 (5)	−0.0004 (5)
C7	0.0209 (7)	0.0244 (8)	0.0230 (8)	−0.0042 (6)	0.0059 (6)	−0.0039 (6)
C8	0.0184 (7)	0.0237 (8)	0.0320 (10)	−0.0030 (6)	0.0058 (7)	−0.0053 (7)
C9	0.0209 (7)	0.0183 (7)	0.0259 (9)	−0.0030 (5)	0.0028 (6)	−0.0027 (6)
C10	0.0365 (12)	0.0214 (9)	0.0426 (13)	0.0023 (8)	−0.0043 (10)	0.0032 (8)
C11	0.0302 (9)	0.0223 (8)	0.0231 (9)	−0.0048 (7)	−0.0050 (7)	0.0024 (6)
C12	0.0340 (11)	0.0256 (9)	0.0310 (11)	−0.0052 (8)	−0.0019 (8)	0.0084 (8)
C13	0.0276 (9)	0.0293 (9)	0.0222 (9)	0.0031 (7)	0.0034 (7)	0.0060 (7)
C14	0.0252 (8)	0.0312 (9)	0.0165 (7)	−0.0003 (7)	−0.0006 (6)	0.0002 (6)
C15	0.0216 (7)	0.0252 (8)	0.0191 (8)	−0.0037 (6)	−0.0001 (6)	0.0002 (6)
C16	0.0294 (10)	0.0324 (11)	0.0360 (12)	−0.0016 (8)	0.0013 (9)	−0.0034 (9)
C17	0.0310 (10)	0.0351 (11)	0.0354 (12)	−0.0064 (9)	0.0124 (9)	−0.0079 (9)
C18	0.0419 (13)	0.0323 (11)	0.0257 (10)	−0.0095 (9)	0.0050 (9)	−0.0009 (8)
C19	0.0340 (12)	0.0349 (12)	0.0383 (13)	−0.0018 (9)	−0.0006 (10)	0.0013 (10)
C20	0.0258 (10)	0.0403 (13)	0.0384 (13)	−0.0036 (9)	0.0079 (9)	−0.0017 (10)

### Bis[N-ethyl-N-(2-hydroxyethyl)dithiocarbamato-κ2S,S'](pyridine)zinc(II) pyridine monosolvate (II) Geometric parameters (Å, º)

**Table:**

Zn—N3	2.0611 (16)	C5—H5B	0.9800
Zn—S1	2.3414 (6)	C5—H5C	0.9800
Zn—S3	2.3666 (6)	C7—C8	1.513 (3)
Zn—S4	2.5627 (6)	C7—H7A	0.9900
Zn—S2	2.6140 (6)	C7—H7B	0.9900
S1—C1	1.7357 (18)	C8—H8A	0.9900
S2—C1	1.7168 (19)	C8—H8B	0.9900
S3—C6	1.7388 (18)	C9—C10	1.521 (3)
S4—C6	1.7195 (19)	C9—H9A	0.9900
O1—C3	1.424 (3)	C9—H9B	0.9900
O1—H1O	0.841 (10)	C10—H10A	0.9800
O2—C8	1.425 (3)	C10—H10B	0.9800
O2—H2O	0.839 (10)	C10—H10C	0.9800
N1—C1	1.332 (2)	C11—C12	1.388 (3)
N1—C2	1.472 (2)	C11—H11	0.9500
N1—C4	1.472 (2)	C12—C13	1.386 (3)
N2—C6	1.330 (2)	C12—H12	0.9500
N2—C7	1.471 (2)	C13—C14	1.384 (3)
N2—C9	1.478 (2)	C13—H13	0.9500
N3—C11	1.337 (3)	C14—C15	1.388 (3)
N3—C15	1.338 (2)	C14—H14	0.9500
N4—C20	1.331 (4)	C15—H15	0.9500
N4—C16	1.339 (3)	C16—C17	1.387 (4)
C2—C3	1.528 (3)	C16—H16	0.9500
C2—H2A	0.9900	C17—C18	1.384 (4)
C2—H2B	0.9900	C17—H17	0.9500
C3—H3A	0.9900	C18—C19	1.376 (4)
C3—H3B	0.9900	C18—H18	0.9500
C4—C5	1.518 (3)	C19—C20	1.379 (4)
C4—H4A	0.9900	C19—H19	0.9500
C4—H4B	0.9900	C20—H20	0.9500
C5—H5A	0.9800		
			
N3—Zn—S1	116.78 (5)	S4—C6—S3	117.38 (10)
N3—Zn—S3	110.34 (5)	N2—C7—C8	113.14 (17)
S1—Zn—S3	132.86 (2)	N2—C7—H7A	109.0
N3—Zn—S4	100.14 (5)	C8—C7—H7A	109.0
S1—Zn—S4	98.846 (19)	N2—C7—H7B	109.0
S3—Zn—S4	73.534 (16)	C8—C7—H7B	109.0
N3—Zn—S2	93.26 (5)	H7A—C7—H7B	107.8
S1—Zn—S2	72.621 (17)	O2—C8—C7	112.46 (17)
S3—Zn—S2	104.146 (17)	O2—C8—H8A	109.1
S4—Zn—S2	166.375 (19)	C7—C8—H8A	109.1
C1—S1—Zn	88.79 (6)	O2—C8—H8B	109.1
C1—S2—Zn	80.65 (6)	C7—C8—H8B	109.1
C6—S3—Zn	87.20 (6)	H8A—C8—H8B	107.8
C6—S4—Zn	81.50 (6)	N2—C9—C10	112.35 (18)
C3—O1—H1O	110 (2)	N2—C9—H9A	109.1
C8—O2—H2O	106 (3)	C10—C9—H9A	109.1
C1—N1—C2	121.87 (16)	N2—C9—H9B	109.1
C1—N1—C4	122.92 (16)	C10—C9—H9B	109.1
C2—N1—C4	115.14 (15)	H9A—C9—H9B	107.9
C6—N2—C7	121.79 (16)	C9—C10—H10A	109.5
C6—N2—C9	122.90 (15)	C9—C10—H10B	109.5
C7—N2—C9	115.16 (15)	H10A—C10—H10B	109.5
C11—N3—C15	118.92 (17)	C9—C10—H10C	109.5
C11—N3—Zn	121.12 (13)	H10A—C10—H10C	109.5
C15—N3—Zn	119.77 (13)	H10B—C10—H10C	109.5
C20—N4—C16	117.0 (2)	N3—C11—C12	122.10 (19)
N1—C1—S2	122.69 (14)	N3—C11—H11	119.0
N1—C1—S1	120.38 (14)	C12—C11—H11	119.0
S2—C1—S1	116.93 (10)	C13—C12—C11	119.2 (2)
N1—C2—C3	111.04 (16)	C13—C12—H12	120.4
N1—C2—H2A	109.4	C11—C12—H12	120.4
C3—C2—H2A	109.4	C12—C13—C14	118.50 (19)
N1—C2—H2B	109.4	C12—C13—H13	120.8
C3—C2—H2B	109.4	C14—C13—H13	120.8
H2A—C2—H2B	108.0	C13—C14—C15	119.14 (19)
O1—C3—C2	111.00 (17)	C13—C14—H14	120.4
O1—C3—H3A	109.4	C15—C14—H14	120.4
C2—C3—H3A	109.4	N3—C15—C14	122.16 (19)
O1—C3—H3B	109.4	N3—C15—H15	118.9
C2—C3—H3B	109.4	C14—C15—H15	118.9
H3A—C3—H3B	108.0	N4—C16—C17	122.9 (2)
N1—C4—C5	113.35 (17)	N4—C16—H16	118.5
N1—C4—H4A	108.9	C17—C16—H16	118.5
C5—C4—H4A	108.9	C18—C17—C16	118.8 (2)
N1—C4—H4B	108.9	C18—C17—H17	120.6
C5—C4—H4B	108.9	C16—C17—H17	120.6
H4A—C4—H4B	107.7	C19—C18—C17	118.8 (2)
C4—C5—H5A	109.5	C19—C18—H18	120.6
C4—C5—H5B	109.5	C17—C18—H18	120.6
H5A—C5—H5B	109.5	C18—C19—C20	118.4 (3)
C4—C5—H5C	109.5	C18—C19—H19	120.8
H5A—C5—H5C	109.5	C20—C19—H19	120.8
H5B—C5—H5C	109.5	N4—C20—C19	124.1 (2)
N2—C6—S4	121.85 (14)	N4—C20—H20	117.9
N2—C6—S3	120.76 (14)	C19—C20—H20	117.9
			
C2—N1—C1—S2	−177.90 (14)	Zn—S3—C6—S4	6.11 (10)
C4—N1—C1—S2	−1.3 (3)	C6—N2—C7—C8	90.0 (2)
C2—N1—C1—S1	1.6 (2)	C9—N2—C7—C8	−94.3 (2)
C4—N1—C1—S1	178.23 (14)	N2—C7—C8—O2	67.3 (2)
Zn—S2—C1—N1	170.49 (16)	C6—N2—C9—C10	92.7 (2)
Zn—S2—C1—S1	−9.02 (9)	C7—N2—C9—C10	−82.9 (2)
Zn—S1—C1—N1	−169.57 (15)	C15—N3—C11—C12	0.3 (3)
Zn—S1—C1—S2	9.94 (10)	Zn—N3—C11—C12	175.27 (19)
C1—N1—C2—C3	85.5 (2)	N3—C11—C12—C13	−0.2 (4)
C4—N1—C2—C3	−91.4 (2)	C11—C12—C13—C14	−0.4 (4)
N1—C2—C3—O1	170.55 (16)	C12—C13—C14—C15	0.9 (3)
C1—N1—C4—C5	104.7 (2)	C11—N3—C15—C14	0.3 (3)
C2—N1—C4—C5	−78.4 (2)	Zn—N3—C15—C14	−174.80 (16)
C7—N2—C6—S4	−5.9 (3)	C13—C14—C15—N3	−0.8 (3)
C9—N2—C6—S4	178.76 (15)	C20—N4—C16—C17	0.0 (4)
C7—N2—C6—S3	173.16 (15)	N4—C16—C17—C18	0.9 (4)
C9—N2—C6—S3	−2.2 (3)	C16—C17—C18—C19	−0.8 (4)
Zn—S4—C6—N2	173.37 (16)	C17—C18—C19—C20	−0.1 (4)
Zn—S4—C6—S3	−5.69 (9)	C16—N4—C20—C19	−1.0 (4)
Zn—S3—C6—N2	−172.97 (15)	C18—C19—C20—N4	1.0 (4)

### Bis[N-ethyl-N-(2-hydroxyethyl)dithiocarbamato-κ2S,S'](pyridine)zinc(II) pyridine monosolvate (II) Hydrogen-bond geometry (Å, º)

**Table:**

*D*—H···*A*	*D*—H	H···*A*	*D*···*A*	*D*—H···*A*
O1—H1*O*···O2^i^	0.84 (2)	1.99 (2)	2.817 (2)	167 (3)
O2—H2*O*···N4	0.84 (3)	1.93 (3)	2.753 (3)	167 (3)
C2—H2*B*···S3^ii^	0.99	2.84	3.773 (2)	157
C14—H14···S2^iii^	0.95	2.69	3.443 (2)	137

Symmetry codes: (i) −*x*+1/2, *y*+1/2, −*z*+3/2; (ii) −*x*+3/2, *y*+1/2, −*z*+3/2; (iii) −*x*+1, −*y*+1, −*z*+1.
